# A “Conscious” Loss of Balance: Directing Attention to Movement Can Impair the Cortical Response to Postural Perturbations

**DOI:** 10.1523/JNEUROSCI.0810-24.2024

**Published:** 2024-10-02

**Authors:** Johnny V. V. Parr, Richard Mills, Elmar Kal, Adolfo M. Bronstein, Toby J. Ellmers

**Affiliations:** ^1^Manchester Metropolitan University Institute of Sport, Manchester M1 7EL, United Kingdom; ^2^Department of Health Sciences, College of Health, Medicine, and Life Sciences, Centre for Cognitive and Clinical Neuroscience, Brunel University London, Uxbridge UB8 3PH, United Kingdom; ^3^Department of Brain Sciences, Centre for Vestibular Neurology, Imperial College, London W6 8RP, United Kingdom

**Keywords:** balance, conscious control, EEG, kinetics, N1, perturbation, posture

## Abstract

“Trying too hard” can interfere with skilled movement, such as sports and music playing. Postural control can similarly suffer when conscious attention is directed toward it (“conscious movement processing”; CMP). However, the neural mechanisms through which CMP influences balance remain poorly understood. We explored the effects of CMP on electroencephalographic (EEG) perturbation-evoked cortical responses and subsequent balance performance. Twenty healthy young adults (age = 25.1 ± 5 years; 10 males and 10 females) stood on a force plate-embedded moveable platform while mobile EEG was recorded. Participants completed two blocks of 50 discrete perturbations, containing an even mix of slower (186 mm/s peak velocity) and faster (225 mm/s peak velocity) perturbations. One block was performed under conditions of CMP (i.e., instructions to consciously control balance), while the other was performed under “Control” conditions with no additional instructions. For both slow and fast perturbations, CMP resulted in significantly smaller cortical N1 signals (a perturbation-evoked potential localized to the supplementary motor area) and lower sensorimotor beta EEG activity 200–400 ms postperturbation. Significantly greater peak velocities of the center of pressure (i.e., greater postural instability) were also observed during the CMP condition. Our findings provide the first evidence that disruptions to postural control during CMP may be a consequence of insufficient cortical activation relevant for balance (i.e., insufficient cortical N1 responses followed by enhanced beta suppression). We propose that conscious attempts to minimize postural instability through CMP acts as a cognitive dual-task that dampens the sensitivity of the sensorimotor system for future losses of balance.

## Significance Statement

“Trying too hard” is known to interfere with skilled movement, such as sports and music playing. Postural control can also paradoxically worsen when individuals direct conscious attention toward maintaining balance. Yet, the brain mechanisms underpinning the counterproductive effects of such conscious movement processing (CMP) remain unclear. Here, we show that impaired postural control when engaging in CMP is expressed by a reduction in the evoked cortical signal following a perturbation to balance. These findings imply that conscious attempts to minimize postural instability may act as a cognitive dual-task that dampens the sensitivity of the sensorimotor system for future losses of balance.

## Introduction

When our movements fail us—or when we worry that they might—motor control can become a conscious, effortful process (Masters and Maxwell, 2008). In sport and music instrument playing, this is usually referred to as “trying too hard.” This is especially true for balance, where movement failure can have catastrophic consequences to health. While engaging in such conscious movement processing (CMP) can occasionally be adaptive (Clark, 2015), the control of balance—and of motor skills more generally (Baumeister, 1984; Parr et al., 2023b)—typically suffers if too much conscious attention is directed toward it (Boisgontier et al., 2017; Uiga et al., 2020; Kal et al., 2022). However, the neural mechanisms underpinning the maladaptive effects of CMP upon balance remain unclear.

Following an external balance perturbation, the central nervous system triggers rapid (∼100 ms) brainstem-mediated postural responses (Horak, 2006; Jacobs and Horak, 2007; Welch and Ting, 2008). This is followed by a negative electroencephalographic (EEG) cortical response (the “N1” evoked potential) across the supplementary motor area ∼100–200 ms after perturbation onset (Marlin et al., 2014; Varghese et al., 2017). The N1 is greater when facing larger perturbations (Payne et al., 2019), when a corrective step is required to avoid falling (Payne and Ting, 2020a; Solis-Escalante et al., 2021; Zaback et al., 2023), when a perturbation is unexpected (Adkin et al., 2006), and in individuals with poorer balance abilities (Payne and Ting, 2020b). Researchers have therefore proposed that the N1 acts as an error detection mechanism that is “primed” for (1) detecting center of mass movements that approach one's limits of stability and (2) predicting the need for compensatory (i.e., stepping) behavioral responses (Payne and Ting, 2020a; Solis-Escalante et al., 2021; Zaback et al., 2023).

The N1 can be influenced by “cognitive processes such as greater perceived threat or *attention to balance*, which have the potential to influence subsequent motor control” (Payne and Ting, 2020b). Indeed, decreased cortical N1 amplitudes occur when attention is directed away from balance via a cognitive dual-task (Quant et al., 2004; Little and Woollacott, 2015). In contrast, greater cortical N1 amplitudes occur when stance is perturbed during conditions which are known to increase attention toward balance (e.g., postural threat (Adkin et al., 2008; Zaback et al., 2023). However, these changes in CMP have co-occurred with increases in physiological arousal and/or cognitive loading, making it difficult to isolate the neural mechanisms through which CMP disrupts postural performance. The primary aim of this study is to therefore explore the direct effects of increased CMP on the cortical N1 response and subsequent postural control performance.

Engaging in CMP is thought to increase the general sensitivity of the sensorimotor system (or “vigilance”) to balance (Ellmers et al., 2021; Ellmers and Kal, 2023; Harris et al., 2023) and may therefore influence pre- and postperturbation cortical activities beyond the N1. For example, CMP could drive changes in EEG beta activity, given evidence that lower preperturbation beta supports perceptual sensitivity toward somatosensory signals (Shin et al., 2017; Mirdamadi et al., 2024) and that higher postperturbation beta activity may reflect increased cortical engagement toward balance recovery following the N1 response (Ghosn et al., 2020; Palmer et al., 2021). Engaging in CMP can also evoke heightened EEG alpha activity across the visual cortex (Sherman et al., 2021; Parr et al., 2023a), which may support the vigilance toward somatosensory processing by down-weighting visual processing (Jensen and Mazaheri, 2010; Gallicchio and Ring, 2020). Despite these findings, the specific role of CMP upon beta and alpha activity remains unknown.

We hypothesized that under conditions of increased CMP, we would observe greater cortical N1 amplitudes, lower preperturbation beta power, and greater preperturbation occipital alpha power, when compared with control conditions where no specific attentional instructions are provided. As directing conscious attention to movement is known to disrupt postural control in healthy young adults (Boisgontier et al., 2017), we also predicted that balance would become impaired during conditions of CMP.

## Materials and Methods

### Participants

Twenty neurotypical young adults participated in the experiment (10 females, 10 males; *M* ± SD age = 25.1 ± 5.0 years; height = 173.30 ± 11.17 cm; weight = 74.30 ± 10.81 kg). Sample size estimates were based on the medium (*d* = 0.71) to large effects (*d* = 0.82) reported upon the cortical N1 under conditions that indirectly manipulate CMP [e.g., heightened postural threat (Adkin et al., 2008) and divided attention (Little and Woollacott, 2015]. Assuming a medium-to-large effect size (*d* = 0.71), a minimum sample size of 18 participants was required to yield 80% power with an alpha level of *p* = 0.05 when comparing mean differences between two related groups (calculated using G*POWER software 3.1; Heinrich University Dusseldorf). All participants were free from any neurological disease and had no prior experience of dizziness or balance problems. The experiment was approved by the Manchester Metropolitan University institutional ethics committee (project ID #56055).

### Protocol

Perturbations were delivered via a bespoke moveable platform (80 × 60 cm with an embedded force plate recording at 1,000 Hz; Type 9281B, Kistler Instrument). The platform was driven by an electromagnetic actuator and controlled through custom written software (LabVIEW v19 SP1, National Instruments) via DAQ card (USB-6210, National Instruments). Participants stood on the force plate, with their feet shoulder width apart and their hands on their hips. Foot positioning was marked to ensure consistency between trials and conditions (i.e., participants could return to the same position between trial blocks, or in the event a step was taken as response to the perturbation). During the trials, participants were instructed to fixate on a cross marked on the wall at eye level, 4 m away.

**Figure 1.**
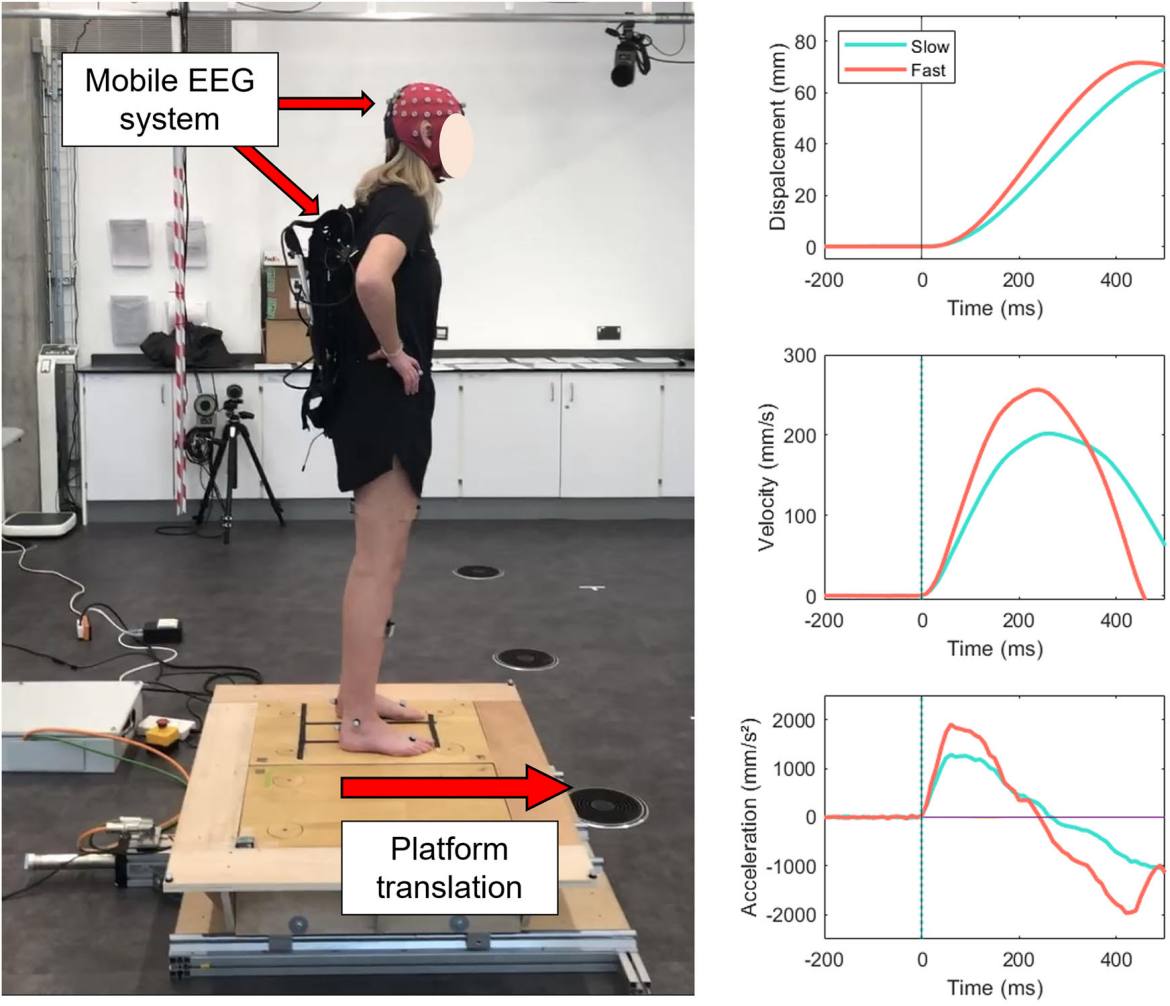
Left, Visual representation of the experimental task. Participants stood with eyes open and feet shoulder width apart on a moveable platform while wearing a mobile EEG system on their back. The platform would translate in the forward direction at two speeds with a consistent displacement. Right, Line plots displaying the displacement, velocity, and acceleration of the initial forward platform translation for each perturbation speed (recorded via motion-capture marker and accelerometer placed on the platform).

Participants experienced two blocks of 50 discrete sine-wave perturbations (7–15 s random delay between each perturbation) consisting of an initial forward translation of the support surface (maximum forward displacement, 70 mm) before reversing direction and completing the sine-wave to return to original position. Each 10 min block consisted of 50 perturbations: 25 fast (0.5 Hz; peak acceleration, 1,883 mm/s^2^; peak acceleration latency, 60 ms) and 25 slow (0.3 Hz; peak acceleration, 1,277 mm/s^2^; peak acceleration latency, 60 ms), presented in a pseudorandom order. For the purpose of this study, we focused only on the initial forward portion of the perturbation (Fig. 1) to not risk contamination of EEG data with the return of the sine-wave perturbation. Perturbations were therefore predictable in amplitude (70 mm max forward displacement) and direction (i.e., forward) but unpredictable in terms of both speed and timing, as perturbations were delivered every 7–15 s. To further maximize the unpredictability of stimulus presentation, participants wore noise-isolating headphones to minimize any anticipatory audio cues. Both perturbation stimuli (fast and slow) were designed to challenge postural stability but small enough to not necessitate a correcting stepping response. To prevent fatigue, participants received a 5–10 min break after each block of trials. To define the onset of platform perturbations, we recorded the kinematics of a reflective marker placed on the platform at a frequency of 100 Hz using a 10-camera motion analysis system (Qualisys v2021.1). The “findpeaks” function in MATLAB was used to identify the forward peaks (i.e., peak forward displacement) in the platform's forward–backward position vector. We then utilized the “ischange” function in MATLAB to identify the moment at which an abrupt change in the vector's acceleration profile first occurred in the 1 s of data prior to each peak.

### Attentional focus manipulation

As we sought to explore how CMP affects the neural control of balance when stance is perturbed, one block (of 50 trials) was performed under conditions designed to induce CMP; while the other block was performed under “control” conditions (no other instructions provided aside from the general task instructions). For the CMP condition, participants were instructed to consciously monitor their postural stability between each perturbation (“focus your attention toward how the weight is distributed beneath your feet”) and minimize any movement in their ankles. These instructions were based on qualitative research that has explored what participants direct their attention toward when CMP (spontaneously) occurs during postural control (Zaback et al., 2016). Prompts and reminders were delivered to ensure that participants maintained this focus of attention throughout the block of trials. The presentation order of conditions (CMP vs Control) was counterbalanced across participants.

After each block of trials, participants completed a four-item questionnaire that assessed the extent to which they directed conscious attention toward their balance during the previous set of trials (e.g., “I am always trying to think about my balance when I am doing this task”; 1 = strongly disagree; 6 = strongly agree; Ellmers and Young, 2018; Ellmers et al., 2021). This questionnaire served as a manipulation check. Scores from the four separate items were summed to produce a total score of state CMP. To assess any carry-over effects (i.e., order effects) of performing the CMP condition first, we performed post hoc independent *t* tests to compare state CMP between participants who performed either the Control or CMP condition first. Results showed no difference between groups for the Control condition (*t*_(18)_ = 0.518; *p* = 0.611), the CMP condition (*t*_(18)_ = 0.767; *p* = 0.453), or the change scores between conditions (*t*_(18)_ = 0.446; *p* = 0.661). After each condition of trials, participants also completed a visual analog scale that ranged from 0 (“not at all anxious”) to 10 (“the most anxious I have ever felt”) to rate the level of state anxiety that they felt during the preceding trials (Castro et al., 2019). Higher scores therefore indicate greater state anxiety. These self-reported assessments were used to confirm that the CMP manipulation led to the intended increase in state CMP, while verifying that any results observed were not confounded by any between-condition differences in state anxiety.

### EEG recording and analyses

The EEG signals were recorded at 1,000 Hz from 29 active shielded AgCl electrodes embedded in a stretchable fabric cap (eego sports, ANT Neuro) positioned according to the extended 10–20 international system (Jurcak et al., 2007). Electrodes in sites CPz and AFz were used as reference and ground, respectively. Nasion, Inion, and preauricular points were used as anatomical landmarks to position the EEG cap. Conductive gel for electrophysiological measurements was used (Signagel, ParkerLabs), and impedance was kept below 20 kΩ. The EEG and force plate (see below) signals were synchronized through a square-wave trigger upon the initiation of an experimental recording.

**Figure 2.**
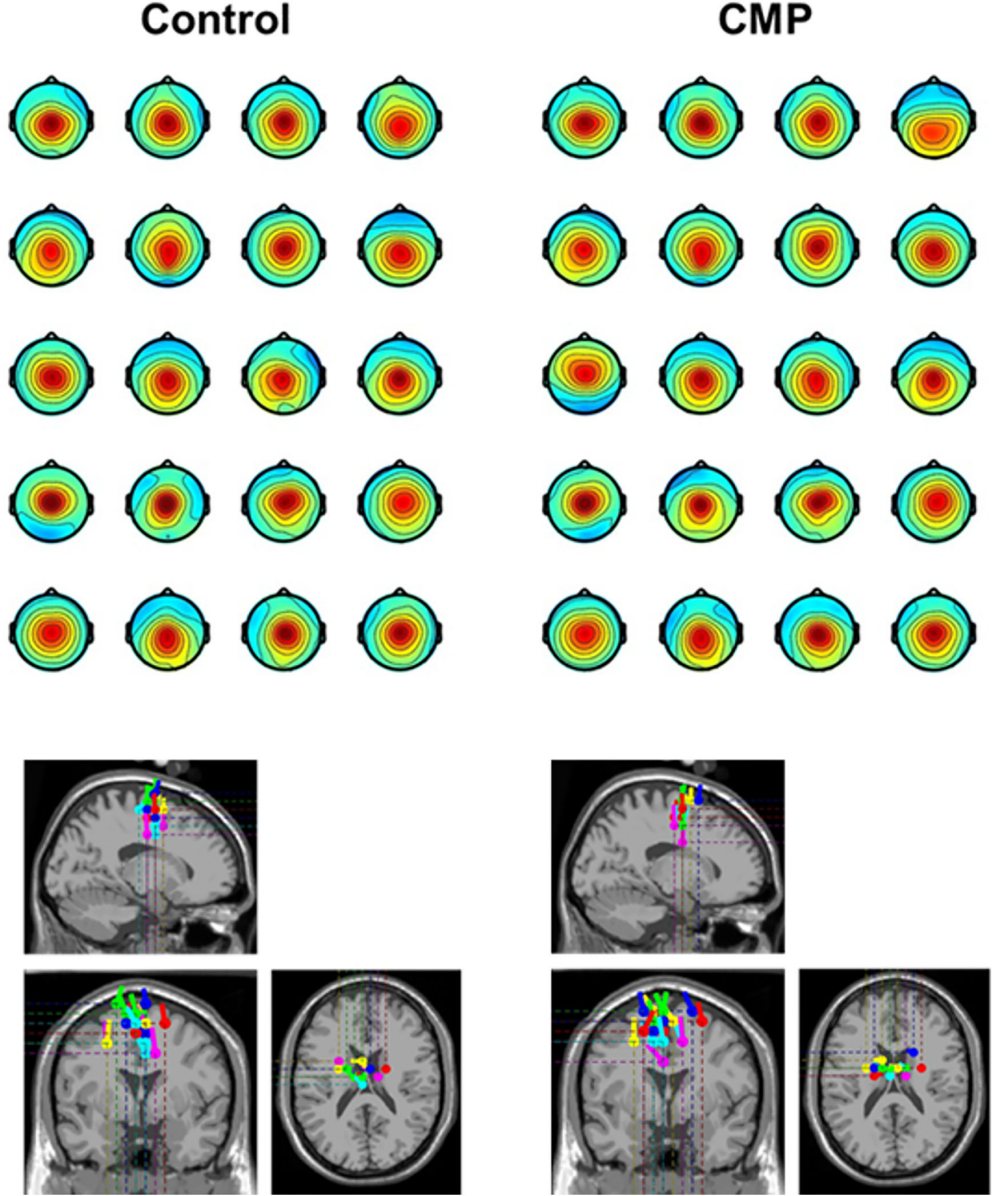
Top, Participant-specific scalp topographies of cortical N1 components for both the Control (top left) and CMP (top right) conditions. Bottom, Cortical N1 sources mapped onto a standard Montreal Neurological Institute (MNI) template and estimated using the DIPFIT plugin. Estimated cortical locations and percentage of power accounted for by the cortical N1 components can be found in Extended Data Table 2-1.

EEG signals were bandpass filtered using the EEGLAB “basic FIR filter (new)” (1–45 Hz, 3,300 filter order, −6 dB cutoff frequency, 1 Hz transition bandwidth) prior to being cut into epochs ranging from −1 to +2 s relative to perturbation onset and rereferenced to the average of all scalp electrodes. These epochs were visually inspected for large EEG contamination from muscular artifacts, but no trials were discarded. No bad EEG channels were identified. Independent component analysis (ICA) weights were obtained separately for each condition through the RunICA infomax algorithm (Jung et al., 1998) running on EEG signals. ICA weights that presented obvious non-neural activity upon visual inspection (e.g., eyeblinks, line noise, muscular artifact) were manually rejected. On average, we retained 25.9 ± 1.1 and 25.9 ± 1.7 components across the CMP and Control conditions, respectively. Following visual inspection, we then identified the brain component that gave rise to a distinct cortical N1. Consistent with other studies, N1 components were localized across the supplementary motor area (Marlin et al., 2014; Varghese et al., 2017), with a midfrontal topography consistent across all participants and across the two experimental conditions (Control and CMP; Fig. 2). For visualization purposes only, cortical N1 sources were further mapped onto a standard MNI template and estimated using the DIPFIT plugin (coarse fit; Oostenveld and Oostendorp, 2002; Klug and Gramann, 2021). Estimated cortical locations and percentage of power accounted for by the cortical N1 components can be found in Extended Data Table 2-1. All processing steps were performed using EEGLAB (v2020.0) functions (Delorme and Makeig, 2004) for MATLAB.

10.1523/JNEUROSCI.0810-24.2024.t2-1 Table 2-1 Characteristics of the primary source contributing to the N1 potential for each participant, including the residual variance (RV %) and the percentage of power accounted for (ppaf %) 100 - 200 ms post perturbation. Download Table 2-1, DOCX file.

#### Preperturbation EEG measures

For preperturbation activity, we extracted single trial spectra from −1000 to 0 ms using Welch's method (non-overlapping windows, 500 window length) for each participant. The FOOOF (Fitting Oscillations & One-Over-F) algorithm (Donoghue et al., 2020) was then used to decompose the trial-level power spectra into aperiodic (1/f) and periodic components (activity above 1/f) from 4 to 30 Hz using the following parameters: max number of peaks, 4; minimum peak height, 0.1; peak threshold, 2; aperiodic module, fixed). Peak periodic beta (15–30 Hz) and peak periodic alpha (8–12 Hz) were extracted from the fitted spectra. If more than one peak was detected, values were averaged across the peaks. Since the width of periodic peaks can vary, we also extracted the area under the spectral curve (AUC; Mirdamadi et al., 2024). As preperturbation beta and alpha oscillatory activities were calculated prior to the perturbation onset, values were averaged across both fast and slow trials within a given condition (CMP vs Control) to increase statistical power. Changes in broadband 1/f activity of the cortical N1 component were also assessed by extracting the aperiodic slope and aperiodic offset using the FOOOF algorithm.

#### Postperturbation EEG analyses

**Figure 3.**
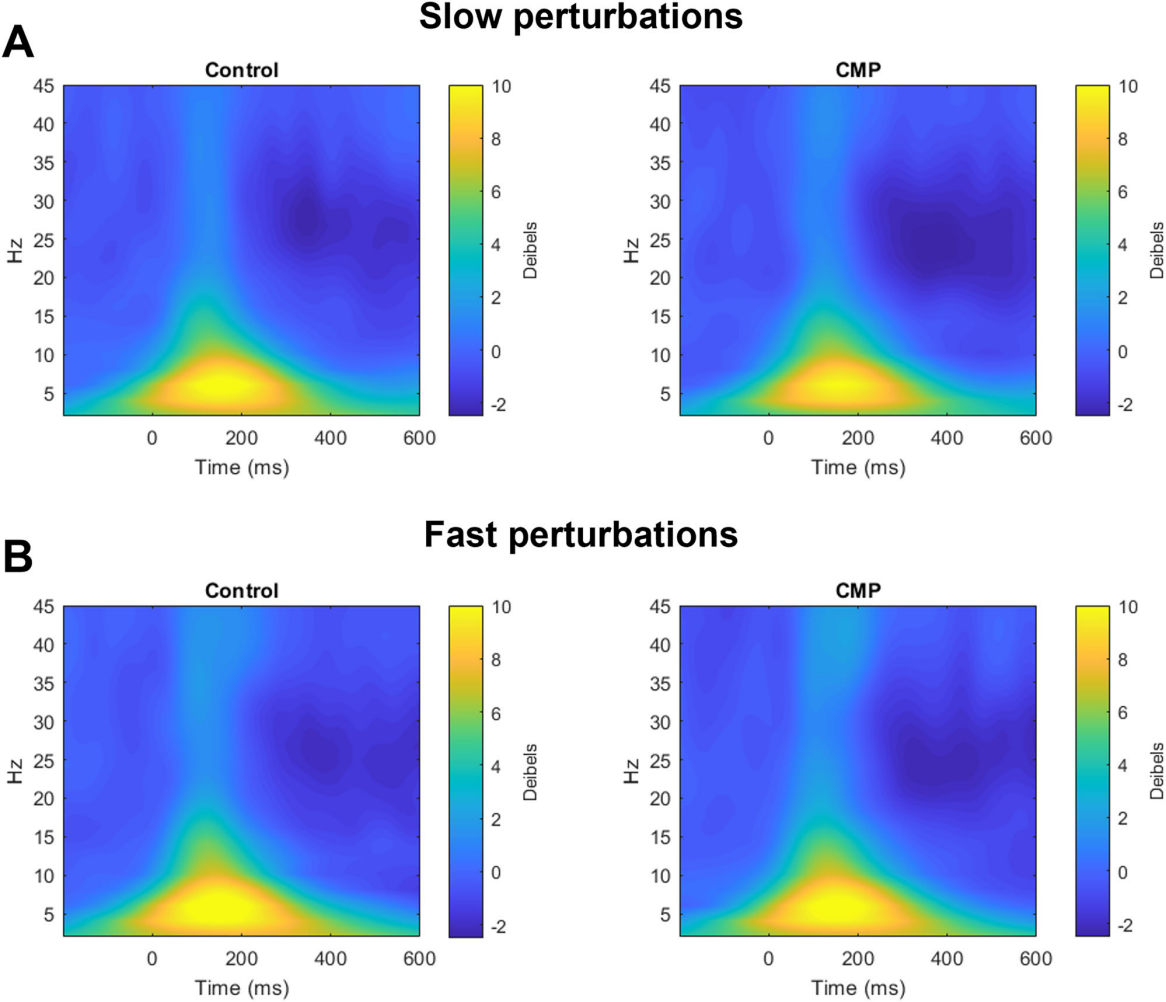
Grand average event-related spectral power of channel Cz across each experimental condition for both slow (***A***) and fast (***B***) perturbations.

To assess the cortical N1 response, we extracted single trial N1 amplitudes from the selected N1 component (Fig. 2). However, given that analytical approaches vary across the literature (with some studies analyzing the N1 component (Solis-Escalante et al., 2021; Mirdamadi et al., 2024) and others focusing only on channel Cz (Varghese et al., 2017; Payne and Ting, 2020b; Zaback et al., 2023), we also performed parallel N1 analyses on channel Cz to confirm whether our findings were robust across component- versus channel-level analyses. Time series data were baseline subtracted (−150 to −50 ms before perturbation onset) for each participant, and the N1 was quantified as the largest negative peak occurring 50–200 ms after perturbation onset. For each participant, N1 amplitudes were subsequently averaged across fast and slow perturbations separately for both the CMP and Control conditions. We also calculated event-related spectral power (ERSP) of both the cortical N1 component- and EEG channel-level data. To achieve this, we performed time–frequency decomposition via trial-by-trial convolution with complex Morlet wavelets. We used 44 frequencies linearly spaced between 2 and 45 Hz, with wavelets logarithmically spaced from 5 to 8 cycles. We then divided decomposed time–frequency data by the average activity from −1000 to −500 ms prior to perturbation across all conditions and trials (i.e., neutral baseline across conditions) before performing a 10*log10 transformation (i.e., decibel change). We then extracted the average beta activity (15–30 Hz) between 200 and 400 ms postperturbation from the selected cortical N1 component as an index of cortical engagement in balance recovery following the cortical N1 response (Ghosn et al., 2020; Palmer et al., 2021). We again performed parallel analyses of postperturbation beta activity on channel Cz to confirm whether our findings were robust across component- versus channel-level analyses. For the purpose of vizualization, grand average ERSP of channel Cz are presented in Figure 3.

### Postural control analyses

We used custom MATLAB scripts to determine the peak velocity of center of pressure (COP) data in response to the initial forward portion of the perturbation. As we used a forward-moving perturbation, we restricted analysis to the anterior-posterior (AP) direction. Peak backward, COP velocity was selected as our outcome variable as it is a direction-specific response to the initial forward perturbation; greater backward CoP velocity generally indicates greater instability and higher risk of falling (Hewson et al., 2010; Masani et al., 2014). First, for each event, we selected and low-pass filtered (5 Hz, second-order bidirectional Butterworth filter) a 3 s AP-COP trace that spanned 2,000 ms preperturbation and 1,000 ms postperturbation. We then corrected this trace for offset using the estimated average AP-COP displacement during the “baseline” period (based on the 1,100–100 ms preperturbation window). Peak velocity of the postural response to the perturbation was then identified as the first negative peak in the derivative of the AP-COP trace in the initial forward portion of the perturbation (Fig. 1). By default, the initial negative peak was selected unless a subsequent peak was of >50% greater magnitude than the earlier peak. The mean latency to peak velocity (termed “peak latency”) for slow perturbations were 219 ms (SD = 29; range, 166–278) and 217 ms (SD = 27; range, 164–271) for Control and CMP conditions, respectively. The mean peak latencies for fast perturbations were 213 ms (SD = 23; range, 172–260) and 212 ms (SD = 21; range, 173–258) for Control and CMP conditions, respectively.

### Statistical analyses

The Gaussian distribution of data were checked via Shapiro–Wilk test of normality. Paired-samples *t* tests were therefore used to determine differences between attention conditions (CMP vs Control) for self-reported conscious processing, self-reported anxiety, preperturbation peak beta and beta AUC, aperiodic exponent, and aperiodic offset. For the N1 amplitude, postperturbation beta activity, and for peak AP-COP velocity, we performed a two-way repeated-measures analysis of variance (ANOVA) with perturbation speed (slow vs fast) and condition (CMP vs Control) as within-subject factors. However, as data for peak AP-COP velocity during the control condition were significantly non-normally distributed (*p *= 0.035), we first performed a log-transformation of AP velocity data prior to ANOVA. Pearson's correlations were then performed to determine any association between N1 amplitude and AP velocity. To explore topographical differences between conditions in preperturbation beta and alpha AUC, we performed channel-wise paired-samples *t* tests (i.e., one *t* test for each channel pair). The multiple-comparisons problem (i.e., one test per channel/pixel) was solved by applying the false discovery rate (FDR) to obtained *p* values. ANOVA effect sizes were reported using partial eta squared (*η_p_*^2^), common indicative thresholds for which are small (0.01), medium (0.06), and large (0.14; Field, 2013). All statistical analyses were performed using IBM SPSS statistics (version 26) with an alpha level of 0.05.

## Results

### Attentional focus manipulation checks

Participants reported directing significantly greater conscious attention toward their balance in the CMP (*M *= 14.50; SD = 4.02) compared with Control condition (*M *= 11.80; SD = 5.45; *t *= −4.61; *p *< 0.001; *d *= 0.56), confirming the effectiveness of the CMP manipulation. There was no difference in state anxiety between conditions, with low levels of anxiety experienced for both (Control, *M *= 1.95, SD = 1.76; CMP, *M *= 1.95, SD = 1.32, *Z *= −0.36, *p *= 0.971, *r *= 0.018).

### N1 amplitude

**Figure 4.**
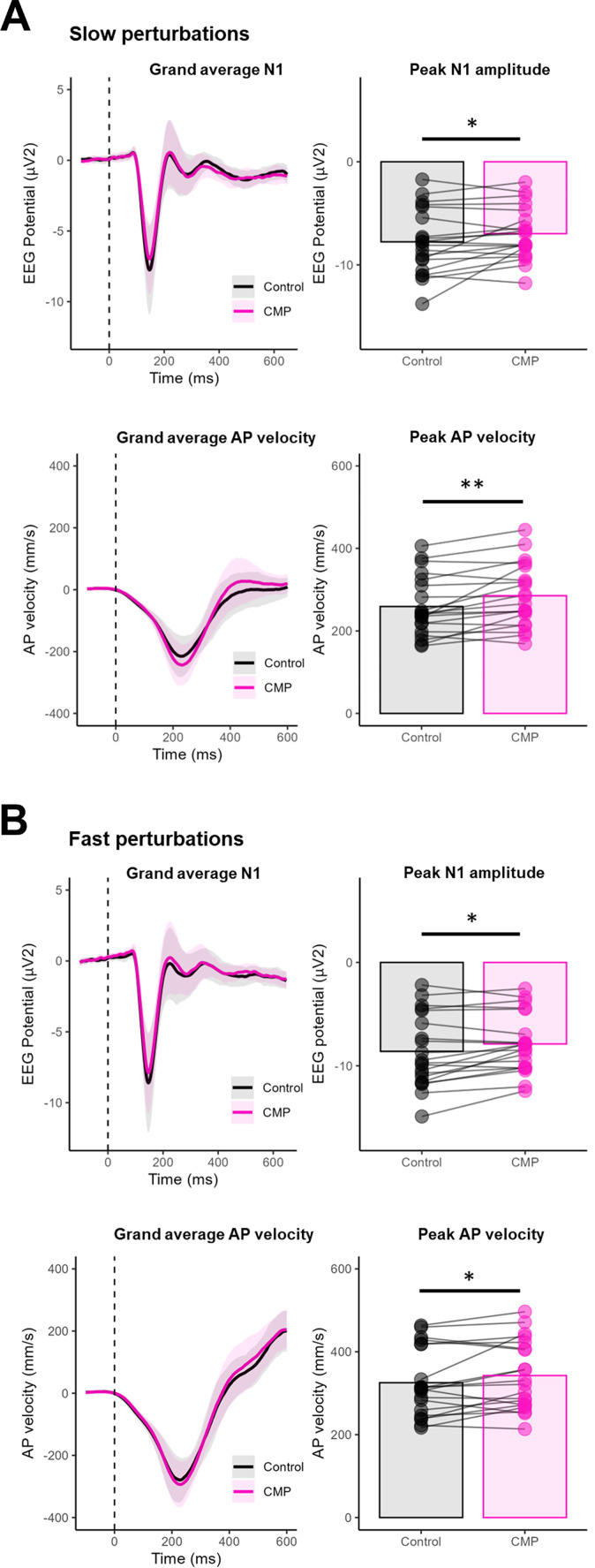
Summary results for the N1 component's ERP and AP velocity for the slow (4***A***; top four panels) and fast perturbations (4***B***; bottom four panels). For each figure, separately presented are the following: top left, Group-level perturbation-evoked potentials, with the thick solid lines and shaded region of the ERP denoting mean and standard deviation, respectively; top right, N1 amplitudes for both the CMP and Control conditions, with the bars denoting group mean values and points denoting individual participant mean values; bottom left, Group-level AP velocity traces for both the CMP and Control conditions, with thick solid lines and shaded region denoting mean and standard deviation, respectively; and bottom right, AP peak amplitudes, with the bars denoting group mean values and points denoting individual participant mean values. For all panels on the right, lines connect the mean values for each participant from the CMP to the Control condition. Asterisks denote a pairwise significant difference at the *p* < 0.05* and *p* < 0.01** levels. A detailed comparison of descriptive and inferential statistics of the cortical N1 amplitude derived from the component and channel Cz analyses is provided in Extended Data Figures 4-1 and 4-2 and in Extended Data Tables 4-1 and 4-2.

Analysis of the cortical N1 component showed a significant main effect of perturbation speed, *F*_(1,19)_ = 28.86, *p *< 0.001, *η_p_*^2^ = 0.603, with larger N1 amplitudes observed during fast compared with slow perturbations (irrespective of attentional focus condition). There was also a significant main effect of Attention condition, *F*_(1,19)_ = 6.11, *p *= 0.023, *η_p_*^2^ = 0.243, with smaller N1 amplitudes observed in CMP compared with the Control condition (irrespective of the perturbation speed). On average, N1 amplitudes during the CMP condition were 8% smaller for fast perturbations and 10% smaller for slow perturbations, compared with Control. There was no Attention × Speed interaction, *F*_(1,19)_ = 0.12, *p *= 0.737, *η_p_*^2^ = 0.006 (Fig. 4). Consistent findings were observed when analyses were performed on channel Cz (rather than the N1 component). However, N1 amplitudes for channel Cz were approximately three times larger than the amplitudes of the N1 component (Extended Data Fig. 4-1). Individual N1 amplitudes from both the component and channel Cz analyses were also highly correlated (*r*s > 0.92), confirming the robustness of the results across component- and channel-level analyses (Extended Data Fig. 4-2). A detailed comparison of descriptive and inferential statistics from the component and channel Cz analyses is provided in Extended Data Tables 4-1 and 4-2.

10.1523/JNEUROSCI.0810-24.2024.f4-1 Figure 4-1 Mean (± SD) N1 amplitudes derived from the component (A) and channel Cz (B) analyses. Jitter points reflect individual participant means. Download Figure 4-1, TIF file.

10.1523/JNEUROSCI.0810-24.2024.f4-2 Figure 4-2 Scatter plots displaying the linear relationship between N1 amplitudes derived from component versus channel Cz analyses for both slow (top row) and fast (bottom row) perturbations and across the Control (left column) and CMP (right column) conditions. Download Figure 4-2, TIF file.

10.1523/JNEUROSCI.0810-24.2024.t4-1 Table 4-1 Mean (± SD) N1 amplitudes derived from both the selected cortical N1 component and channel Cz. Download Table 4-1, DOCX file.

10.1523/JNEUROSCI.0810-24.2024.t4-2 Table 4-2 Statistical results derived from a Condition (Control vs CMP) x Perturbation Speed (Slow vs Fast) repeated measures ANOVA comparing the cortical N1 amplitude derived from both the selected cortical N1 component and channel Cz. Asterisks denote significant main effects at the p < .05 (*), p < .01 (**), and p < .001 (***) level. Download Table 4-2, DOCX file.

### Postural control

**Figure 5.**
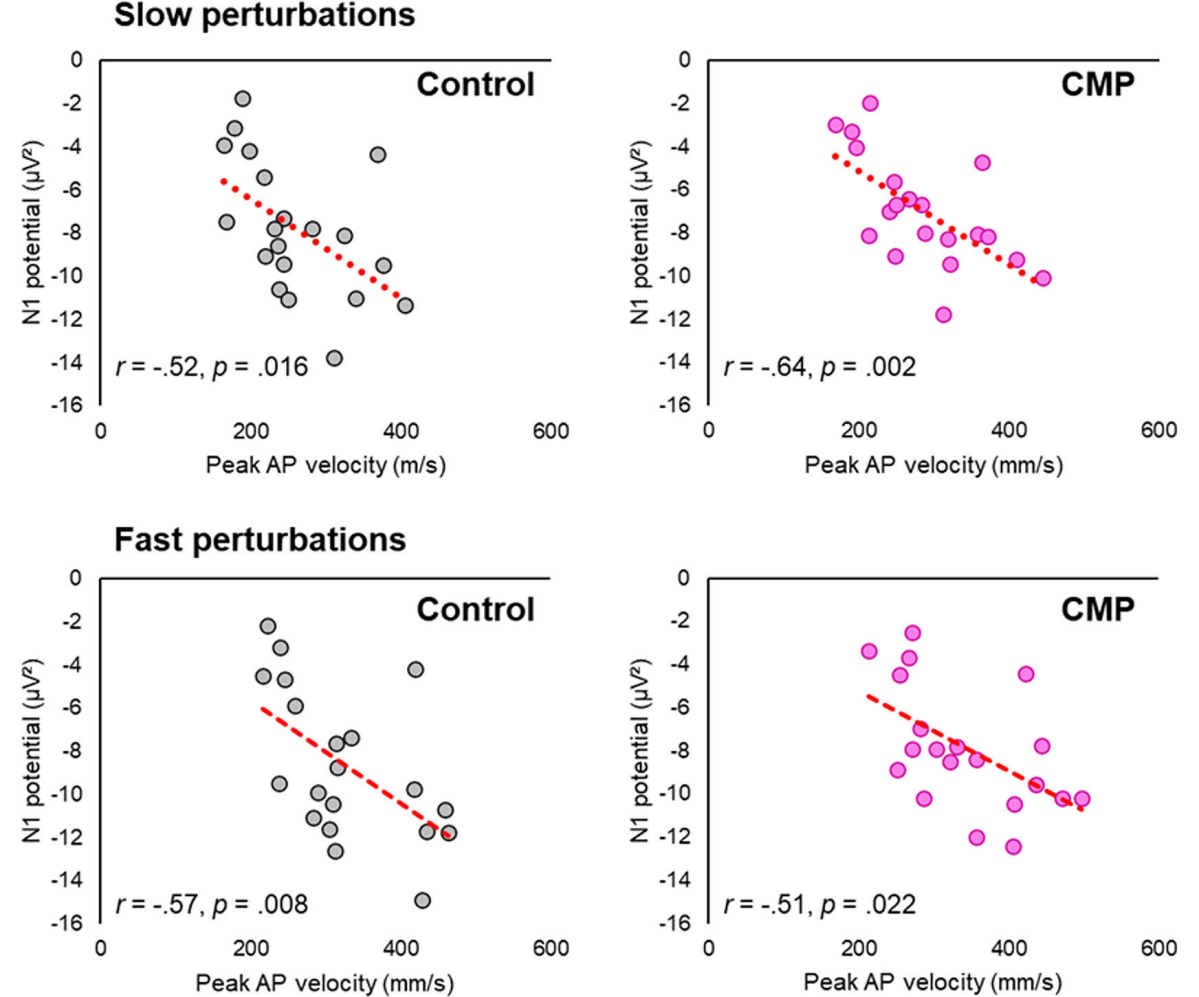
Scatterplots denoting the Pearson's correlation between the amplitude of the N1 component and peak AP velocity for both slow (top row) and fast (bottom row) perturbations.

There was a significant main effect of perturbation Speed (*F*_(19)_ = 274.683; *p *< 0.001; *η_p_*^2^ = 0.935), with greater peak AP velocities observed for fast compared with slow perturbations. There was also a significant main effect of Attention condition (*F*_(1,19)_ = 7.915; *p *= 0.011; *η_p_*^2^ = 0.294) and a significant interaction between Attention and perturbation Speed (*F*_(1,19)_ = 9.109; *p *= 0.007; *η_p_*^2^ = 0.324). Post hoc comparisons showed peak AP velocities to be significantly greater during the CMP condition compared with the Control condition for both fast (*p *= 0.047) and slow (*p *= 0.004) perturbations, with this effect more pronounced for the slow perturbations (Fig. 4). For fast perturbations, Pearson's correlations also revealed a significant negative correlation between peak AP velocity and N1 amplitude for both the CMP (*r *= −0.51; *p *= 0.022) and Control conditions (*r *= −0.57; *p *= 0.008), whereby greater velocities were associated with smaller N1 amplitudes. The same relationship was observed for slow perturbations during both the CMP (*r *= −0.64, *p *= 0.002) and Control (*r *= −0.52, *p *= 0.016) conditions (Fig. 5).

#### Pre- and postperturbation cortical activity

**Figure 6.**
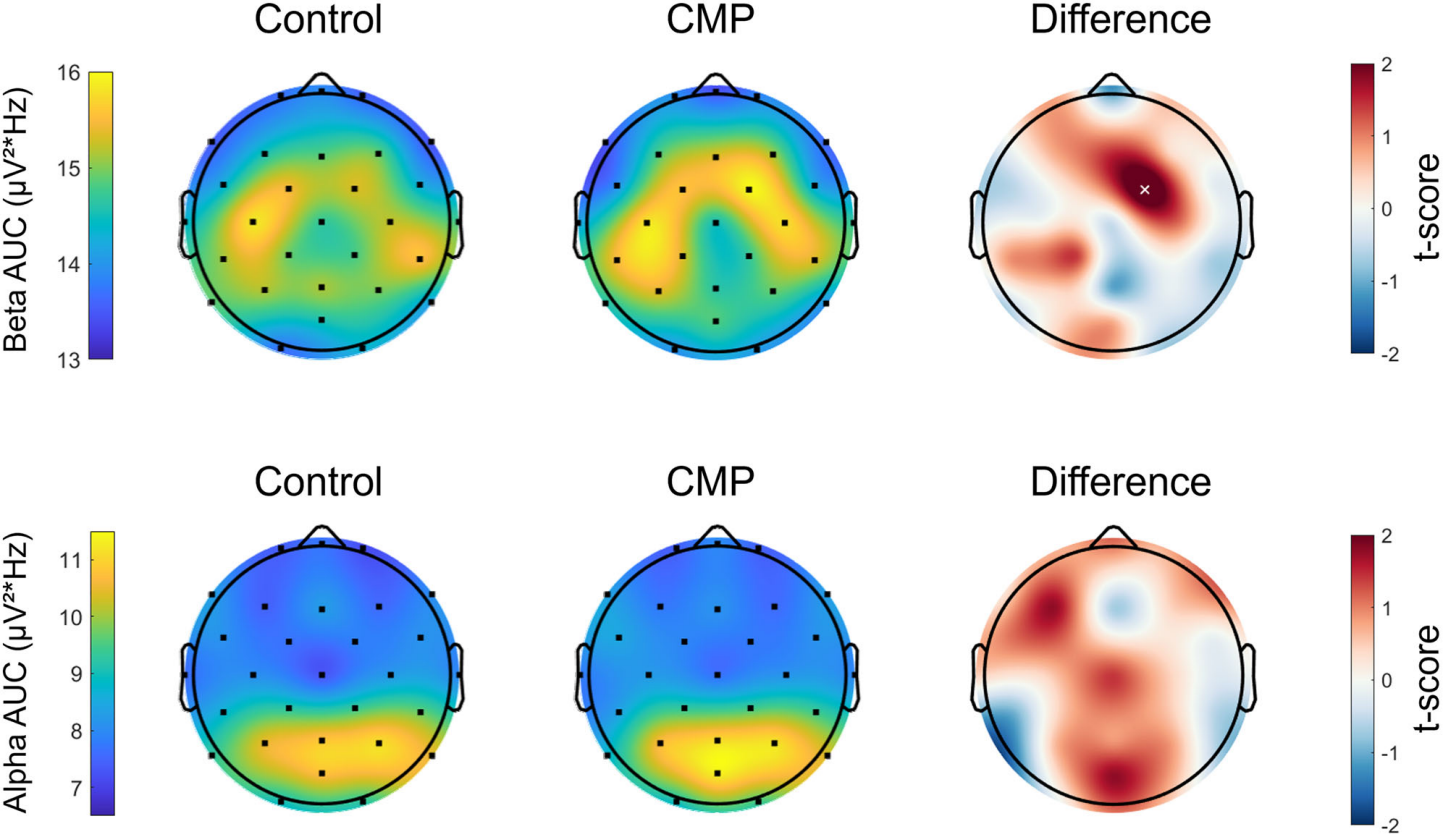
Scalp maps denoting the group mean values of preperturbation beta AUC (top row) and alpha AUC (bottom row) for the Control and CMP conditions, presented as normalized area under the spectral curve. The scalp maps furthest right denote the *t* scores obtained through channel-wise paired comparisons, with red regions indicating greater power in the CMP compared with Control condition, and blue regions indicating greater power in the Control compared with CMP condition. The white ‘x’ denotes a significant difference in beta AUC between channels at channel FC2 prior to FDR statistical corrections.

**Figure 7.**
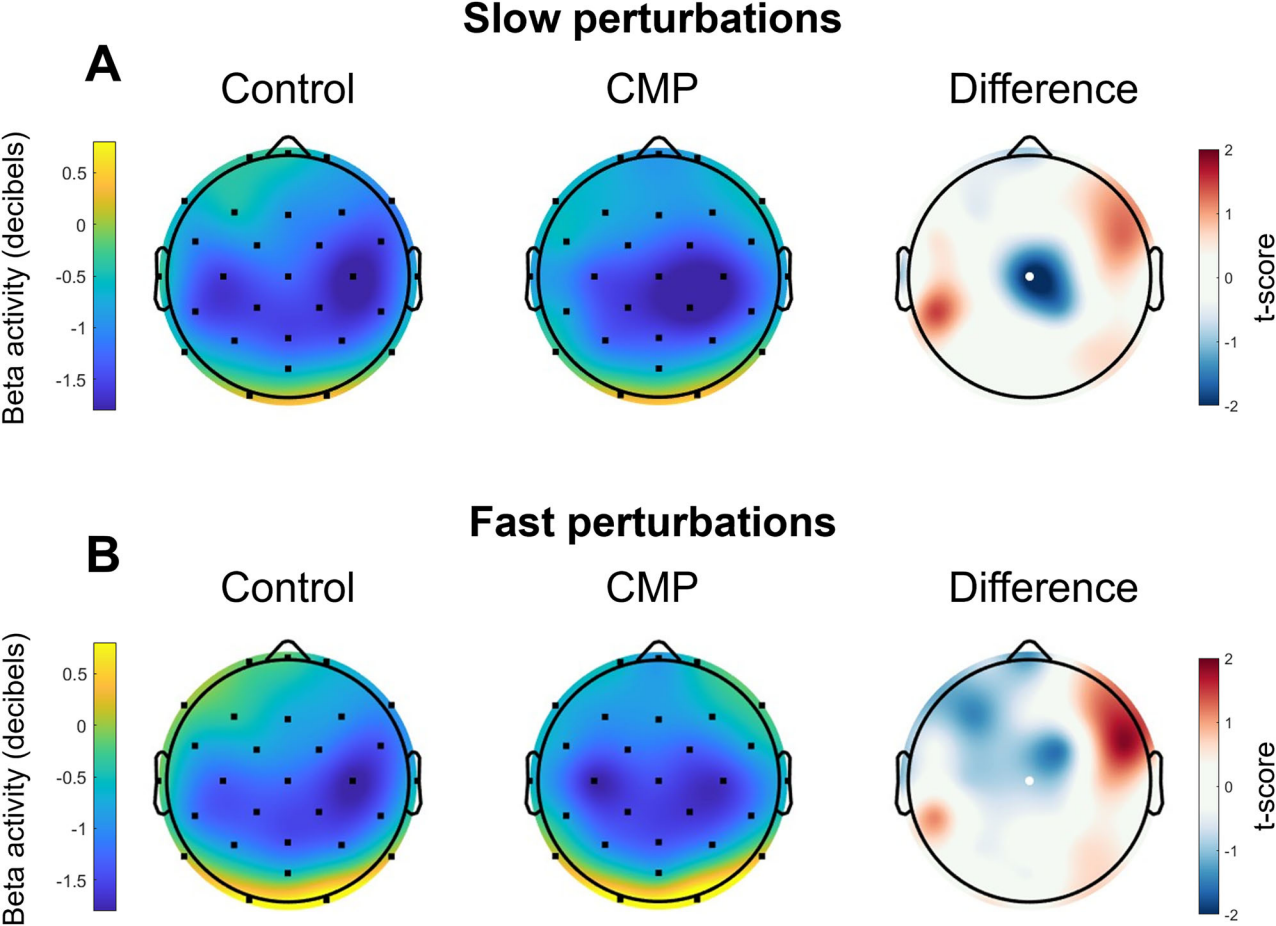
Scalp maps denoting the group-mean postperturbation beta activity (decibels) across conditions for both the slow (top row) and fast (bottom row) perturbations. The scalp maps further right denote the *t* scores obtained through channel-wise comparisons, with red regions indicating higher beta activity in the CMP compared with Control condition, and blue regions indicating lower beta activity in the CMP compared with the Control condition. Channel Cz is indicated by the white dot, as this channel was the focus of these particular analyses.

Paired *t* tests revealed no difference in the cortical N1 component’s preperturbation peak beta (*t*_(19)_ = 0.14, *p* = 0.891, *d* = 0.03), beta AUC (*t*_(19)_ = 0.24, *p* = 0.817, *d* = 0.05), aperiodic exponent (*t*_(19)_ = 0.69, *p* = 0.498, *d* = 0.15), or aperiodic offset (*t*_(19)_ = 1.49, *p* = 0.150, *d* = 0.34) between CMP and Control conditions. Topographical analyses of preperturbation periodic activity revealed no channel-wise differences between conditions for peak alpha, peak beta, or alpha AUC. Higher preperturbation beta AUC was initially observed for the CMP condition compared to the Control condition for channel FC2 (*t*_(19)_ = 2.63, *p* = 0.016, *d* = 0.59), but this effect failed to reach significance following FDR corrections (*p* = 0.060; Fig. 6). For postperturbation beta activity of the N1 component, the ANOVA revealed no main effect of Condition, *F*_(1,19)_ = 2.31, *p *= 0.144, *η_p_*^2^ = 0.109; no main effect of perturbation Speed, *F*_(1,19)_ = 3.71, *p *= 0.069, *η_p_*^2^ = 0.163; and no Condition × Speed interaction, *F*_(1,19)_ = 0.01, *p *= 0.976, *η_p_*^2^ = 0.000. However, for postperturbation beta activity of channel Cz, the ANOVA showed a significant main effect of Condition, *F*_(1,19)_ = 4.45, *p *= 0.048, *η_p_*^2^ = 0.190, with lower beta activity during the CMP condition compared with the Control condition, particularly for the slower perturbations (Fig. 7). There was neither a significant main effect of perturbation Speed, *F*_(1,19)_ = 1.44, *p *= 0.244, *η_p_*^2^ = 0.071, nor Condition × Speed interaction, *F*_(1,19)_ = 3.68, *p *= 0.070, *η_p_*^2^ = 0.162.

## Discussion

We explored how directing conscious attention toward balance affects the cortical control of posture during discrete perturbations to quiet stance. Our findings revealed that the cortical N1—a neural signal involved in monitoring postural instability and mobilizing compensatory balance-correcting responses (Payne and Ting, 2020a; Solis-Escalante et al., 2021; Zaback et al., 2023)—was significantly smaller during conditions of experimentally induced CMP. Behaviorally, this was coupled with greater peak COP velocity during the CMP condition, indicating greater postural instability. Although effective postural control requires some degree of attentional resources (Woollacott and Shumway-Cook, 2002; Boisgontier et al., 2017), directing too much attention toward balance can disrupt postural control—much like how athletic performance breaks down when experts adopt a self-focus (Baumeister, 1984; Smith et al., 2003; Parr et al., 2023a). The present findings provide the first evidence that such maladaptive effects of CMP on postural control appear to be expressed by insufficient activation at the cortex relevant for postural control.

Previous work has reported larger N1 signals during conditions of increased postural threat (Adkin et al., 2008; Zaback et al., 2023) and reduced N1s when performing a cognitive dual-task (Quant et al., 2004; Little and Woollacott, 2015). Although not a direct manipulation of CMP, individuals will reliably direct greater conscious attention toward movement when their balance is threatened and they become anxious/fearful about falling (Huffman et al., 2009; Zaback et al., 2016; Ellmers et al., 2023). Conversely, individuals will direct less attention toward balance during conditions of dual-task (Johnson et al., 2020; Ellmers et al., 2021). We therefore expected larger N1 amplitudes under conditions of CMP that was induced independent of postural threat and that this result would reflect an increased sensitivity of the sensorimotor system for responding to postural disturbances during self-focused attention (Harris et al., 2023). Self-report data confirmed that our manipulation was successful at isolating CMP from perceived threat/anxiety. However, contrary to our prediction, we observed significantly smaller N1 responses under conditions of CMP. This reduction in N1 amplitudes (average reduction of ∼9%) is akin to reductions previously reported during conditions of cognitive dual-task (between ∼5 and 20% reduction; Quant et al., 2004; Little and Woollacott, 2015). This suggests that the larger N1 amplitudes observed previously during conditions of increased postural threat (which is known to induce CMP) may instead reflect threat-related increases in mental vigilance or arousal, rather than changes in attention to movement (Zaback et al., 2023). Indeed, emotional arousal has also been shown to modulate the amplitude of the N1 in non-motor (i.e., cognitive) tasks (Luna et al., 2023).

Researchers have proposed that the N1—which is localized to the supplementary motor area—acts as an instability and/or error detection mechanism that is “primed” for (1) detecting center of mass movements that approach one's limits of stability and (2) mobilizing compensatory stepping responses (Payne and Ting, 2020a; Solis-Escalante et al., 2021; Zaback et al., 2023). Supporting this stance, the present findings showed that the cortical N1 scales with perturbation intensity, with greater N1 amplitudes observed during the fast (compared with slow) perturbation. Our findings also revealed larger N1 amplitudes in individuals with poorer within-task balance performance (i.e., greater peak COP velocity; Fig. 5), which aligns with previous work showing larger N1 responses in individuals with poorer generalized balance ability (Payne and Ting, 2020b). Collectively, these findings support the notion that the cortical N1 amplitude reflects the allocation of cognitive resources toward compensatory balance-correcting responses (Payne and Ting, 2020a). The reduction in N1 amplitudes observed during conditions of CMP therefore likely reflects a maladaptive process. Indeed, on group level, these reductions were accompanied by disruptions in postural performance (increased peak COP velocity—and hence greater disturbance—in response to the perturbation). We are unable to draw causal inferences between the reduction in N1 and the subsequently disrupted postural control in the present work. However, as the N1 occurred on average 68 ms (SD = 24 ms) before peak instability (Fig. 4, grand averages), the neural processes underpinning the N1 response may have directly influenced subsequent balance performance.

CMP, by definition, is a “conscious” process, meaning that it requires attentional resources (Ellmers and Young, 2018). Engaging in this form of motor control can therefore act like a cognitive dual-task and limit the resources available for processing other tasks or information (Uiga et al., 2018; Parr et al., 2023a). During the CMP condition, participants were instructed to consciously monitor their postural stability and minimize ankle movement during the preperturbation period. We suggest therefore that individuals were so focused on consciously minimizing instability during the preperturbation period that they became less able to flexibly shift attentional resources toward processing the perturbation itself, resulting in a maladaptively smaller N1 and disrupted postural response. In other words, conscious attempts to maximize stability prior to a loss of balance acts like a cognitive dual-task that reduces the attentional resources available for processing the instability and then behaviorally responding once the loss of balance itself occurs (Quant et al., 2004; Little and Woollacott, 2015). We therefore propose that conscious attempts to minimize postural instability in a given moment serves to dampen the sensitivity of the sensorimotor system for future losses of balance, via disruptions to the “central set” (the nervous system's ability to prepare itself for upcoming sensory information and movement; Horak et al., 1989).

However, the effect of CMP upon the cortical N1 may differ across balance-impaired populations for whom CMP reflects a compensatory strategy to overcome poorer (and less “automatic”) balance (Clark, 2015; Boisgontier et al., 2017; Kal et al., 2022). For instance, it is possible that older adults with fear of falling may instead use CMP proactively in a way that enhances, rather than disturbs, the central set (Ellmers et al., 2023). Future work should therefore look to extend these findings beyond healthy young adults. Nonetheless, these findings provide the evidence that, in neurotypical young adults with relatively good balance control, CMP may disrupt postural control via insufficient compensatory activation at the cortex in response to perturbations.

Contrary to our prediction, the CMP manipulation had no effect on preperturbation oscillatory alpha or beta activity. Within the context of balance, lower preperturbation beta EEG activity of the cortical N1 component is associated with enhanced perception of the subsequent perturbation to balance (Mirdamadi et al., 2024), suggesting that lower beta activity may reflect a more sensitive sensory processing system. Given that CMP is proposed to increase perceptual sensitivity for postural disturbances (Ellmers et al., 2021; Harris et al., 2023), we had expected CMP would thus lower preperturbation beta. In line with previous research (Sherman et al., 2021; Parr et al., 2023a), we had also expected CMP to promote elevated alpha activity across the visual cortex, possibly reflecting a mechanism that supports vigilance to somatosensory processing by down-weighting visual processing through regional inhibition (Jensen and Mazaheri, 2010). However, no differences in preperturbation alpha or beta activity were observed, which suggests that our CMP manipulation did not alter ongoing perceptual sensitivity prior to postural disturbances. That said, there was some evidence that CMP appeared to increase oscillatory beta activity across the pre-motor region (i.e., channel FC2); although this effect failed to reach significance following statistical corrections. Future research should clarify CMP’s role in modulating preparatory neural mechanisms during postural challenges by developing more targeted hypotheses, increasing statistical power, and potentially applying less conservative correction methods.

Previous research has also reported higher post-N1 beta activity in individuals with poorer balance (Palmer et al., 2021), and when experiencing larger perturbations (Ghosn et al., 2020), suggesting a (conscious) compensatory role for such neural activity. However, we instead observed significantly larger reductions in postperturbation beta activity during CMP irrespective of perturbation size. While the functional role of sensorimotor beta oscillations is still not fully understood (Spitzer and Haegens, 2017; Barone and Rossiter, 2021), researchers have proposed that reductions in beta activity during an ongoing action may reflect a “decrease in somatosensory responsiveness for the efficient unfolding of the movement” (p. 22, Kilavik et al., 2013). The reduced beta activity we observed during the late recovery phase of the perturbation could therefore reflect a continued dampening of the sensorimotor system (i.e., beyond the initial cortical N1 response) when engaging in CMP. Previous researchers have consistently proposed CMP to enhance, rather than dampen, sensorimotor sensitivity during postural control (Ellmers et al., 2021; Harris et al., 2023), but our findings question this interpretation of CMP. It is also important to note that this finding was restricted to the channel-level (i.e., Cz) analyses, suggesting these postperturbation features were not captured by the single component that contributes to the cortical N1. Future research should look to further scrutinize the specific mechanisms through which CMP alters postperturbation beta activity.

### Conclusion

Our findings revealed that directing conscious attention toward balance significantly reduced the size of the cortical N1. As this was coupled with poorer postural control, this reduced cortical response is likely maladaptive in nature. We therefore provide evidence that the maladaptive effects of CMP upon balance may be driven by insufficient activation at the cortex relevant for postural control. We propose that conscious attempts to minimize postural instability in a given moment acts as a cognitive dual-task that serves to dampen the sensitivity of the sensorimotor system for future losses of balance. These findings provide novel insight into the neural mechanisms underpinning the maladaptive behavioral effects of “trying too hard” during motor performance.

### Data Availability

The data and code associated with this study are publicly available on the Open Science Framework (at https://doi.org/10.17605/OSF.IO/F9TMP).
