# Supplementary material for: A “Conscious” Loss of Balance: Directing Attention to Movement Can Impair the Cortical Response to Postural Perturbations
Source: J Neurosci. 2024 Oct 2;44(48):e0810242024. doi: 10.1523/JNEUROSCI.0810-24.2024 (PMC11604137; doi:10.1523/JNEUROSCI.0810-24.2024)
Supplement: Table 2-1 — Characteristics of the primary source contributing to the N1 potential for each participant, including the residual variance (RV %) and the percentage of power accounted for (ppaf %) 100 - 200 ms post perturbation. Download Table 2-1, DOCX file. [file jneuro-44-e0810242024-s001.docx]

**Table 2-1.** Characteristics of the primary source contributing to the N1 potential for each participant, including the residual variance (RV %) and the percentage of power accounted for (ppaf %) 100 – 200 ms post perturbation.

|  |  |  |  |  |  | ppaf (%) | |
| --- | --- | --- | --- | --- | --- | --- | --- |
| ID | x | y | z | RV (%) | Location | CMP | Control |
| 1 | -11.09 | -11.09 | 69.55 | 4.02 | Superiorparietal R | 62.73 | 73.07 |
| 2 | -11.09 | -11.09 | 61.82 | 4.63 | Superiorparietal R | 79.28 | 78.67 |
| 3 | -3.70 | -11.09 | 61.82 | 1.75 | Superiorparietal R | 84.61 | 77.23 |
| 4 | -25.87 | 3.70 | 46.36 | 5.69 | Superiorparietal L | 29.79 | 55.29 |
| 5 | 3.70 | -3.70 | 38.64 | 3.18 | Precentral R | 72.26 | 68.00 |
| 6 | -25.87 | -3.70 | 46.36 | 3.18 | Superiorparietal R | 75.91 | 81.44 |
| 7 | 3.70 | -3.70 | 54.09 | 1.46 | Postcentral R | 68.22 | 82.78 |
| 8 | 3.70 | -3.70 | 54.09 | 1.33 | Postcentral R | 80.88 | 86.11 |
| 9 | -3.70 | -11.09 | 54.09 | 1.78 | Superiorparietal R | 64.18 | 75.52 |
| 10 | 3.70 | -3.70 | 61.82 | 1.10 | Postcentral R | 73.25 | 80.18 |
| 11 | -3.70 | -18.48 | 61.82 | 9.75 | Superiorparietal R | 38.96 | 37.35 |
| 12 | 3.70 | -3.70 | 61.82 | 0.97 | Postcentral R | 81.88 | 89.16 |
| 13 | -18.48 | -3.70 | 77.27 | 5.05 | Superiorparietal R | 62.44 | 56.11 |
| 14 | 3.70 | -3.70 | 77.27 | 14.05 | Postcentral R | 43.28 | 53.08 |
| 15 | 18.48 | -3.70 | 61.82 | 1.72 | Precentral R | 88.11 | 85.63 |
| 16 | 11.09 | -11.09 | 38.64 | 1.60 | Precentral R | 93.97 | 96.88 |
| 17 | 3.70 | -3.70 | 46.36 | 0.88 | Postcentral R | 88.11 | 86.53 |
| 18 | -3.70 | 3.70 | 61.82 | 5.54 | Postcentral L | 68.26 | 75.70 |
| 19 | -3.70 | -11.09 | 69.55 | 4.59 | Superiorparietal R | 7.95 | 11.46 |
| 20 | -3.70 | -11.09 | 61.82 | 7.19 | Superiorparietal R | 71.88 | 82.03 |
