# Supplementary figures and images for: A “Conscious” Loss of Balance: Directing Attention to Movement Can Impair the Cortical Response to Postural Perturbations
Source: J Neurosci. 2024 Oct 2;44(48):e0810242024. doi: 10.1523/JNEUROSCI.0810-24.2024 (PMC11604137; doi:10.1523/JNEUROSCI.0810-24.2024)

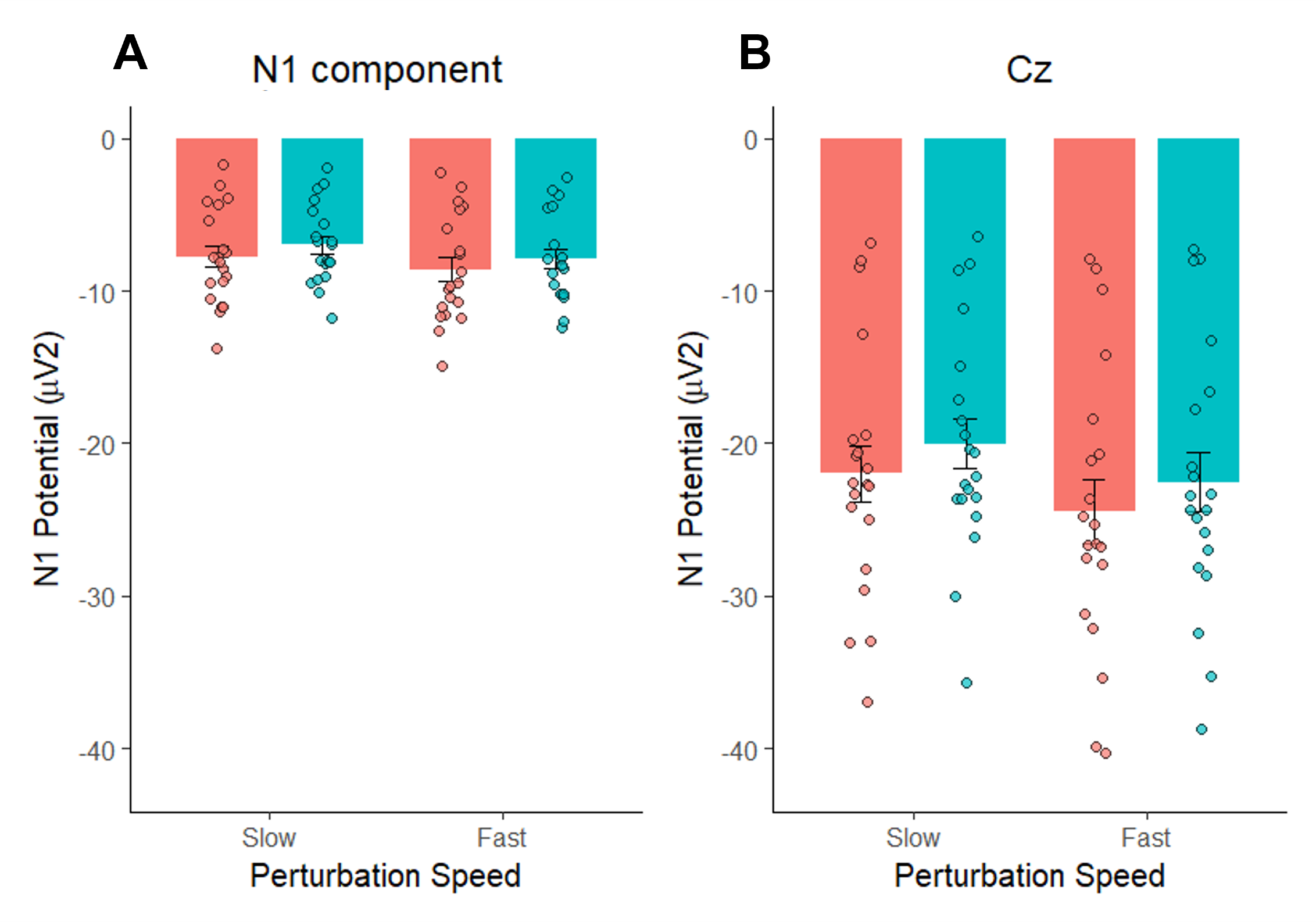

Supplement: Figure 4-1 — Mean (± SD) N1 amplitudes derived from the component (A) and channel Cz (B) analyses. Jitter points reflect individual participant means. Download Figure 4-1, TIF file. [file jneuro-44-e0810242024-s002.tif]

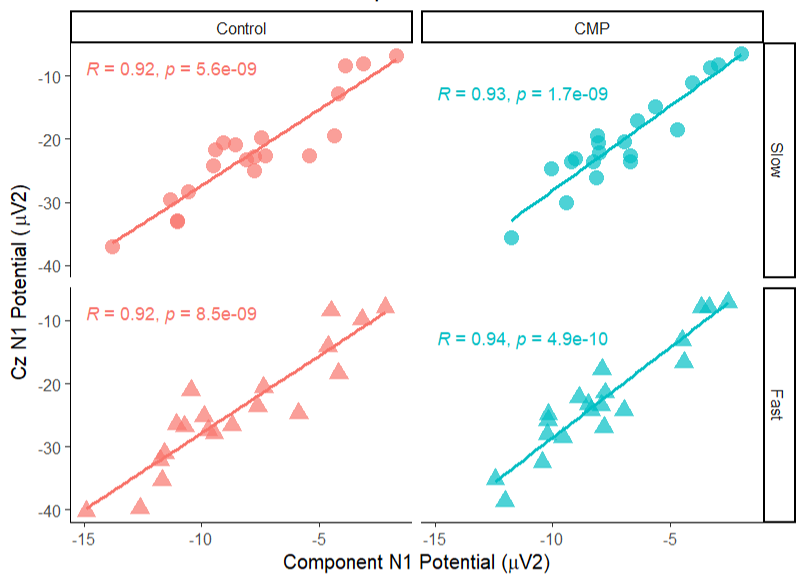

Supplement: Figure 4-2 — Scatter plots displaying the linear relationship between N1 amplitudes derived from component versus channel Cz analyses for both slow (top row) and fast (bottom row) perturbations and across the Control (left column) and CMP (right column) conditions. Download Figure 4-2, TIF file. [file jneuro-44-e0810242024-s003.tif]
