# Supplementary material for: A “Conscious” Loss of Balance: Directing Attention to Movement Can Impair the Cortical Response to Postural Perturbations
Source: J Neurosci. 2024 Oct 2;44(48):e0810242024. doi: 10.1523/JNEUROSCI.0810-24.2024 (PMC11604137; doi:10.1523/JNEUROSCI.0810-24.2024)
Supplement: Table 4-1 — Mean (± SD) N1 amplitudes derived from both the selected cortical N1 component and channel Cz. Download Table 4-1, DOCX file. [file jneuro-44-e0810242024-s004.docx]

**Table 4-1.** Mean (± standard deviation) N1 amplitudes derived from both the selected cortical N1 component and channel Cz.

|  | **Control** | | **CMP** | |
| --- | --- | --- | --- | --- |
|  | **Slow** | **Fast** | **Slow** | **Fast** |
| **N1 component** | -7.76 ± 3.16 | -8.60 ± 3.51 | -6.97 ± 2.67 | -7.88 ± 2.86 |
| **Channel Cz** | -21.98 ± 8.23 | -24.42 ± 9.33 | -20.02 ± 7.36 | -22.52 ± 8.70 |
