# Supplementary material for: A “Conscious” Loss of Balance: Directing Attention to Movement Can Impair the Cortical Response to Postural Perturbations
Source: J Neurosci. 2024 Oct 2;44(48):e0810242024. doi: 10.1523/JNEUROSCI.0810-24.2024 (PMC11604137; doi:10.1523/JNEUROSCI.0810-24.2024)
Supplement: Table 4-2 — Statistical results derived from a Condition (Control vs CMP) x Perturbation Speed (Slow vs Fast) repeated measures ANOVA comparing the cortical N1 amplitude derived from both the selected cortical N1 component and channel Cz. Asterisks denote significant main effects at the p < .05 (*), p < .01 (**), and p < .001 (***) level. Download Table 4-2, DOCX file. [file jneuro-44-e0810242024-s005.docx]

**Table 4-2.** Statistical results derived from a Condition (Control vs CMP) x Perturbation Speed (Slow vs Fast) repeated measures ANOVA comparing the cortical N1 amplitude derived from both the selected cortical N1 component and channel Cz. Asterisks denote significant main effects at the *p* < .05 (*), *p* < .01 (**), and *p* < .001 (***) level.

|  | **Condition** | **Speed** | **Interaction** |
| --- | --- | --- | --- |
| **N1 component** | *p* = .023, ƞp2 = .243* | *p* < .001, ƞp2 = .603** | *p* = .737, ƞp2 = .006 |
| **Channel Cz** | *p* = .010, np2 = .298* | *p* < .001, np2 = .548*** | *p* = .939, np2 = .000 |
